# To err is human, to correct is public health: a systematic review examining poor quality testing and misdiagnosis of HIV status

**DOI:** 10.7448/IAS.20.7.21755

**Published:** 2017-08-29

**Authors:** Cheryl C. Johnson, Virginia Fonner, Anita Sands, Nathan Ford, Carla Mahklouf Obermeyer, Sharon Tsui, Vincent Wong, Rachel Baggaley

**Affiliations:** ^a^ Department of HIV, World Health Organization, Geneva, Switzerland; ^b^ Department of Clinical Research, London School of Hygiene and Tropical Medicine, London, UK; ^c^ Department of Psychiatry and Behavioral Sciences, Medical University of South Carolina, Charleston, SC, USA; ^d^ Department of Essential Medicines and Health Products, World Health Organization, Geneva, Switzerland; ^e^ Department of Epidemiology and Population Health, American University of Beirut, Beirut, Lebanon; ^f^ Department of International Health, Johns Hopkins University, Bloomberg School of Public Health, Baltimore, MD, USA; ^g^ US Agency for International Development, Washington, DC, USA

**Keywords:** HIV, HIV testing, misdiagnosis, misclassification, diagnostic error, false positive, healthcare, patient safety

## Abstract

**Introduction**: In accordance with global testing and treatment targets, many countries are seeking ways to reach the “90-90-90” goals, starting with diagnosing 90% of all people with HIV. Quality HIV testing services are needed to enable people with HIV to be diagnosed and linked to treatment as early as possible. It is essential that opportunities to reach people with undiagnosed HIV are not missed, diagnoses are correct and HIV-negative individuals are not inadvertently initiated on life-long treatment. We conducted this systematic review to assess the magnitude of misdiagnosis and to describe poor HIV testing practices using rapid diagnostic tests.

**Methods**: We systematically searched peer-reviewed articles, abstracts and grey literature published from 1 January 1990 to 19 April 2017. Studies were included if they used at least two rapid diagnostic tests and reported on HIV misdiagnosis, factors related to potential misdiagnosis or described quality issues and errors related to HIV testing.

**Results**: Sixty-four studies were included in this review. A small proportion of false positive (median 3.1%, interquartile range (IQR): 0.4-5.2%) and false negative (median: 0.4%, IQR: 0-3.9%) diagnoses were identified. Suboptimal testing strategies were the most common factor in studies reporting misdiagnoses, particularly false positive diagnoses due to using a “tiebreaker” test to resolve discrepant test results. A substantial proportion of false negative diagnoses were related to retesting among people on antiretroviral therapy.

**Conclusions**: HIV testing errors and poor practices, particularly those resulting in false positive or false negative diagnoses, do occur but are preventable. Efforts to accelerate HIV diagnosis and linkage to treatment should be complemented by efforts to improve the quality of HIV testing services and strengthen the quality management systems, particularly the use of validated testing algorithms and strategies, retesting people diagnosed with HIV before initiating treatment and providing clear messages to people with HIV on treatment on the risk of a “false negative” test result.

## Introduction

In the last decade, HIV testing services have been scaled-up substantially. In 2005, it was estimated that only 12% of people who wanted an HIV test were able to access testing; and that only 10% of people with HIV in Africa knew their status [1]. In contrast, between 2010 and 2014, more than 600 million people in 122 low- and middle-income countries received HIV testing [2], and as of 2015, approximately 60% of people with HIV were aware of their status [3]. Such scale-up has been possible through the expansion of provider-initiated testing and counselling and community-based testing programmes, which have routinized HIV testing and extended services to many people.

Rapid diagnostic tests (RDTs) have been instrumental to the scale-up of HIV testing, particularly in resource-limited settings where access to laboratory services is poor. RDTs have been shown to be highly accurate and can often provide a same-day diagnosis when used within a validated testing strategy (i.e. the order in which the tests are performed) and algorithm (i.e. the exact tests used within the testing strategy) according to high (≥5%) and low HIV prevalence (<5%), as recommended by the World Health Organization (WHO) [4–6]. Recent reports, however, have shown that HIV testing is not always conducted appropriately [7,8], and in some countries, quality systems have not kept pace with testing scale-up. According to a review of national HIV testing policies, less than 20% of testing strategies were consistent with WHO guidance, and only two included recommendations on retesting prior to the initiation of antiretroviral therapy (ART) [9]. In some cases, poor-quality testing has resulted in incorrect test results and the misdiagnosis of HIV status [10–14].

HIV misdiagnosis refers to any testing event where a diagnosis is missed, inappropriately delayed or incorrect (either false positive or false negative) [15]. Poor-quality HIV testing and misdiagnosis have negative consequences for individuals, families, communities, health workers and health services. False negative diagnoses represent missed opportunities to identify an HIV infection and link people to early treatment. False positive diagnoses may cause social and emotional harm and create mistrust of health workers and the test results they deliver. Without addressing HIV testing quality, new guidance offering same-day treatment to all people diagnosed with HIV [16] could lead to inappropriate ART initiation [11]. Once individuals are on treatment, because ART reduces antibody production and can cause seroreversion, for example, false negative test results, determining a person’s true HIV status can be especially challenging [17,18].

We conducted this systematic review to assess the magnitude of misdiagnosis and to identify and describe poor HIV testing practices using RDTs, including those which may have led to incorrect test results and misdiagnosis.

## Methods

We systematically searched for peer-reviewed articles published from 1 January 1990 to 19 April 2017 using a predefined search strategy in the following electronic databases: PubMed, CINAHL and EMBASE. All conferences of the International AIDS Society were searched from July 2001 through July 2016; the most recent Conference on Retroviruses and Opportunistic Infections (2014–2017) database were searched because past conference abstracts were unavailable. Conferences of the African Society of Laboratory Medicine (ASLM) were searched 2012–2016, as well as the ASLM website and other key global health websites (see supplementary information). We searched reference lists to identify additional literature. This process was repeated until no new citations were identified. Experts were also contacted to identify additional reports. No geographic restrictions were placed on the search, but the review was limited to studies published in English.

Studies were eligible if they used at least two RDTs and reported on HIV misdiagnosis, factors related to potential misdiagnosis or described quality issues and related to HIV testing error.

Initial titles were screened by one investigator (VF) to determine eligibility. A second and a third screening was then carried out (VF, ST and CJ). All differences were resolved through consensus. Data from all sources were extracted and placed into standardized forms and verified in duplicate (VF and ST). CJ and NF assessed study quality (see supplementary data).

Potential factors relating to misdiagnosis were extracted from studies using defined categories: (a) clerical error (error in documenting and reporting information essential to a correct status); (b) user error (operator error collecting specimen, performing an HIV RDT or interpreting the result); (c) suboptimal testing strategy (errors related to the order in which specific RDTs are used, also known as a testing strategy); (d) poor management and supervision (lack of active quality management systems); (e) weak reactive results (faint lines appearing on test strips); and (f) additional factors including cross-reactivity, acute/early infection and testing among people on ART.

Other summary measures included: misdiagnosis rates (total number of false positive diagnoses reported over the total number of HIV-positive tests retested and reported using a specific testing algorithm and the total number of false negative diagnoses reported over the number of HIV-negative tests retested and reported using a specific testing algorithm). For studies exclusively among people diagnosed with HIV, reporting on false positive statuses, the total study population was used as the denominator.

For each study, rates of diagnostic error and misdiagnosis and corresponding 95% confidence intervals (CIs) were calculated, using Wilson’s approach, and this was displayed graphically using forest plots [19–21]. All statistical analyses were conducted in STATA v13.0.

## Results

**Figure 1.**
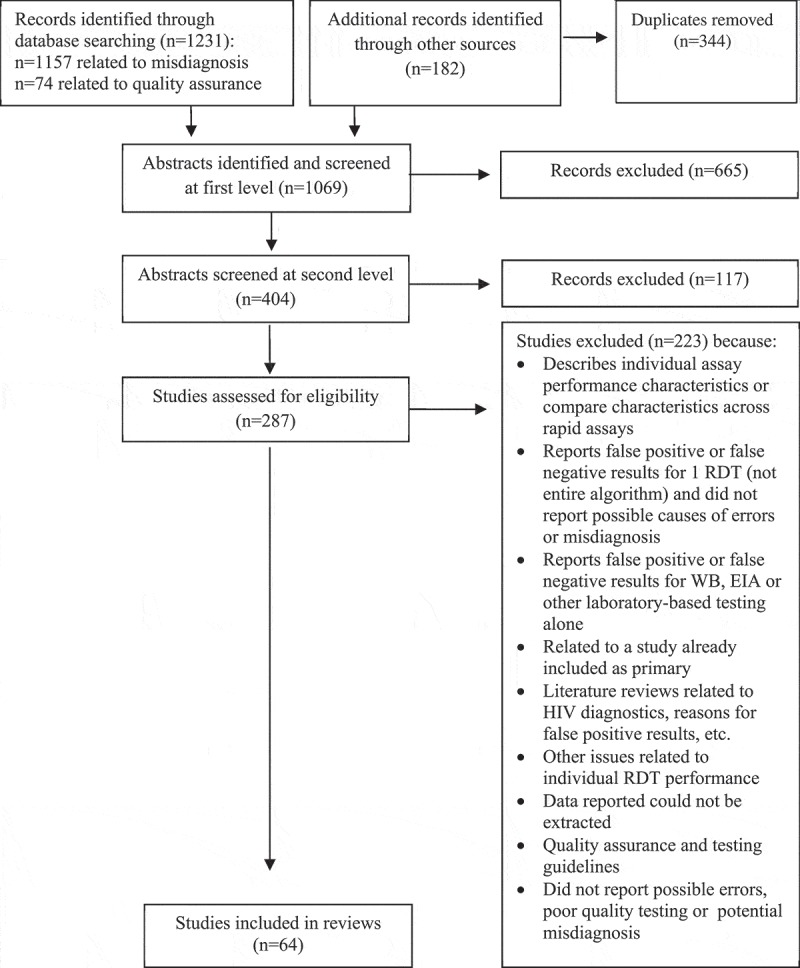
Study selection process. RDT: rapid diagnostic test; WB: Western blot; EIA: enzyme immunoassay.

Sixty-four studies reporting on misdiagnosis of HIV and factors potentially related to misdiagnosis were included in this review (Figure 1 and Table 1).

**Table 1.** Classification of included studies (*n* = 64)

Category	Study	Location
**Potential HIV misdiagnosis and related factors**	Aghokeng et al. [22]	Cameroon
	Baltazar et al. [23]	Mozambique
	Baveewo et al. [24]	Uganda
	Bock et al. [14]	South Africa and Zambia
	Boeras et al. [25]	Zambia and Rwanda
	CDC [26]	Low- and low–middle-income countries (not specified)
	Crucitti et al. [27]	Benin, India, South Africa, Uganda and India
	da Costa et al. [28]	Brazil
	Eller et al. [29]	Uganda
	Fogel et al. [23]	Multiple countries in Africa
	Galiwango et al. [30]	Cameroon
	Granade et al. [31]	USA
	Gray et al. [32]	Uganda
	Hsiao et al. [33]	South Africa
	Jentsch et al. [34]	South Africa, Tanzania, Uganda and Zambia
	Kanal et al. [35]	Cambodia
	Karugaba et al. [36]	Uganda
	Khan et al. [37]	Swaziland
	Klarkowski et al. [38]	Central Africa Republic, Congo, DRC, Ethiopia, Haiti, India, Cote d’Ivoire, Myanmar, Uganda and Zimbabwe
	Klarkowski et al. [10]	DRC
	Kufa et al. [39]	South Africa
	Learmonth et al. [40]	Multi-country study (26 countries)
	Manak et al. [16]	Nigeria
	Maparo et al. [41]	Zimbabwe
	Martin et al. [42]	USA
	Masina et al. [43]	Malawi
	Mayaphi et al. [44]	South Africa
	Mehra et al. [45]	India
	Mine et al. [46]	Botswana
	Nelson et al. [47]	Mozambique
	Sacks et al. [48]	UK
	Shanks et al. [11]	DRC, Burundi and Ethiopia
	Shanks et al. [13]	Ethiopia
	Shanks et al. [12]	Ethiopia
	Simoncini et al. [49]	Niger
	Stetler et al. [50]	Honduras
	Tchounga et al. [51]	Burkina Faso, Cote d’Ivoire and Mali
	Wolpaw et al. [52]	South Africa
	Viani et al. [53]	USA and Mexico
	Young et al. [54]	Mozambique
**Focus on misdiagnosis of HIV-negative serostatus**	Bassett et al. [55]	South Africa
	Kahemele et al. [56]	Tanzania
	Matambo et al. [57]	Zimbabwe
	Olaru et al. [58]	Zimbabwe
**General quality issues from sites conducting HIV testing services**	Adebayo et al. [59]	Nigeria
	Benzaken et al. [60]	Brazil
	Bile et al. [61]	Botswana
	Cham et al. [62]	30 countries in Africa
**General quality issues**	Iwe et al. [63]	Nigeria
**from sites conducting**	Kalou et al. [64]	Uganda and Tanzania
**HIV testing services**	Kitheka et al. [65]	Kenya
*(Continued)*	Kyaw et al. [8]	Myanmar
	Louis et al. [66]	Haiti
	Lali et al. [67]	Uganda
	Manyazewal et al. [68]	Ethiopia
	Mashauri et al. [69]	Tanzania
	Mwangala et al. [70]	Zambia
	Ntim et al. [71]	Ghana
	Ocheng et al. [72]	Tanzania
	Plate et al. [5]	11 countries in Africa
	SEAD [7]	South Africa
	Sushi et al. [73]	India
	Tegbaru et al. [74]	Ethiopia

DRC: Democratic Republic of Congo.

Most studies were carried out in Africa (*n* = 48) [5,7,10–14,16,22–25,29,30,32–34,36,37,39,41,43,44,46,47,49,51,52,54–59,61–65,67–72,74,75], followed by in the Americas (*n* = 7) [28,31,42,50,53,60,66], Asia (*n* = 4) [8,35,45,73] and Europe (*n* = 1) [48]. There were also four multi-country/regional studies [26,27,38,40]. Samples varied by size and unit of measurement, including clients (*n* = 38 studies, range: 303,010 to 1 clients), specimens (*n* = 15 studies, range: 9419 to 16 specimens), health workers performing HIV tests (*n* = 5 studies, range: 3835 to 39 personnel) and sites where HIV testing was performed (*n* = 12 studies, range: 602 to 4 sites). Nine studies reported more than one unit of measure, and three studies did not specify sample size (see supplementary information). The majority of studies occurred in a facility-based setting; studies carried out in community settings included the workplace (*n* = 1) [57], home-based testing (*n* = 2) [14,39] and a mobile setting (*n* = 1) [32].

### Factors related to the quality of HIV testing and potential misdiagnosis

Several factors, including HIV testing errors, were reported frequently (*n* = 131 times) across all included studies (see Table 2).

**Table 2.** Reported HIV testing errors and factors potentially related to misdiagnosis

Category	No. of Studies
Incorrect/suboptimal testing strategy or algorithm (e.g. testing strategies not aligned to the World Health Organizationrecommendations, such as a tiebreaker or parallel testing strategies, use of a single RDT to make an HIV-positivediagnosis)	37
User error (e.g. errors performing RDT or interpreting results, misapplication of buffer, inaccurate reading time and otherhuman errors)	25
Poor or inadequate management and supervision (e.g. work load stress, staff shortages, lack of training, poor adherenceto testing strategy or testing algorithm, substandard operating procedures, testing in window period)	21
Other factors (e.g. acute infection, cross-reactivity, known HIV status/on ART)	18
Clerical/technical errors (e.g. mislabelling, poor record-keeping and clerical mistakes)	16
Weak reactive test results (e.g. faint or ghost lines appearing on test strip)	14

RDT: rapid diagnostic test; ART: antiretroviral therapy. Table includes 63 reporting studies. One study (Bile et al. 2017) did not report a specific factor or error related to misdiagnosis. Some studies reported multiple factors related to poor quality testing and factors that could be related to potential misdiagnosis.

**Table 3.** **Rates of false positive diagnosis rates among people diagnosed with HIV and/or enrolled in care or antiretroviral therapy (ART)**

Study/author	Sample size	Total no. of retested	No. of false positives	Percentage of false positive diagnoses
Klarkowski et al. 2009	365	229	24	6.6
Shanks et al. 2013c	914	54	44	4.8
Shanks et al. 2013b	149	149	7	4.7
Shanks et al. 2013a	78	78	2	2.6
Khan et al. 2017	2533	88	14	0.55
Hsiao et al. 2017	952	37	3	0.3
Maparo et al. 2015	1447	1447	4	0.28
Nelson et al. 2016	3160	3146	3	0.1

Thirty-seven studies reported using a suboptimal testing strategy that differed from the WHO recommendations [5,8,11–14,16,22–30,32–34,36–39,42–44,49,51,53,59,62,64–66,68,72,75]. Suboptimal testing strategies included using a highly specific first-line test and highly sensitive second-line test [14,33,39,55], using a single RDT for HIV-positive diagnoses [11,66,72], using a high prevalence testing strategy in a low prevalence setting [16,49], using a parallel testing algorithms and a tiebreaker testing strategy (where a third assay is used to resolve discrepant test results and rule in HIV infection) [5,12,13,16,24,25,27–30,32,34,36,37,68].

User errors, incorrectly performing the test procedure or incorrectly interpreting results, defined as human errors, were reported in 25 studies [7,8,11,14,26–28,31,33,34,37,40,42,46,52,54,57,60,65–68,70,72,73]. Errors identified included users having difficulty with specimen collection [14,28,68], performing RDTs [31,73], interpreting test results [10,24,27,30,32,40,42,48,62,65,66,74], reading test results too early [7] and not using the correct reagents/buffer [7].

Twenty-one studies reported inadequate management and supervision [7,8,11,26,27,31,41,43,46,52,59,62,64–67,69,71,72,74]. Of these, 10 studies reported issues with management of supplies [7,11,26,27,62,64,67,69,72,74], including stock-outs [7,26,62,64,67,69], the use of damaged or expired RDTs [26,27,64,67] and inappropriate RDTs (i.e. syphilis RDTs) for HIV testing [72]. Other factors related to poor management and supervision included testing within the window period without referring clients for retesting [32,45], HIV testing performed by undertrained or ineligible staff [7,31,59,64,72], low levels of retesting to verify diagnosis before ART initiation [43], poor participation in external quality assessment (EQA) schemes [62], poor site-level supervision [65] and poor adherence to standard operating procedures [7,35,52,59,67,69].

Sixteen studies reported clerical errors [8,11,26,28,29,31,34,35,45,50,63–65,67,73,75]. Errors included poor record-keeping [35], data reporting problems, labelling and transcription mistakes [73] and specimen mix-ups. Poor record-keeping, according to one study, resulted in nearly 30% of errors leading to incorrect status [67]. Clerical errors were not always clearly defined and may not have always led to misdiagnosis [28].

Fourteen studies reported challenges related to weak reactive test results, particularly difficulty with interpretation [8,10,24,27,30,32,36,38,40,42,44,48,62,74]. A study, which assessed the proficiency of laboratory technicians, found that specimens with very weak levels of HIV-1/2 antibodies were less accurately reported [40]. In Uganda, two studies found that the majority of false reactive results came from weak reactive RDTs [32,36]. A study from the UK that assessed the visual depiction of false reactive and true positive readings reported that most false reactive specimens had a fainter test line than true positive specimens [48]. Two studies reported incorrect reading of weak reactive bands contributed to the misdiagnosis [10,11].

Eighteen studies reported on several other testing errors and factors potentially related to misdiagnosis. Nine of these studies reported cross-reactivity either between RDTs within an algorithm or with population and individual characteristics [10,22,24,25,27,32,38,56,60]. One study suggested that cross-reactivity between assays used within an algorithm resulted in false positive statuses [27]. Another hypothesized that cross-reactivity may present as weak reactive lines and thereby cause misdiagnosis [32]. Six studies [10,11,25,38,56,60] reported potential issues with RDTs interacting with characteristics of individuals undergoing testing [10,11,38], including having low levels of HIV-1/2 antibodies due to late stage HIV infection [56,60] and exposure of assays to adverse environmental conditions during storage and use [25,38].

Additionally, six studies reported that a proportion of false negative diagnoses were among people with a known HIV status who were on ART [14,16,39,44,47,58]; one of these studies was among children on ART retested using an oral fluid-based HIV RDT [58]. And three studies reported false negative results were due to patients testing in the window period [45] or with acute or early infection [16,44]. For instance, in South Africa, 0.04% (95% CI: 0.0–0.001) and 0.3% (95% CI: 0.1–0.4) of clients with a false negative diagnosis using serology tests were later found to have acute or early HIV infection after retesting with nucleic acid testing technologies [44].

### False positive diagnostic errors and misdiagnosis rates

**Figure 2.**
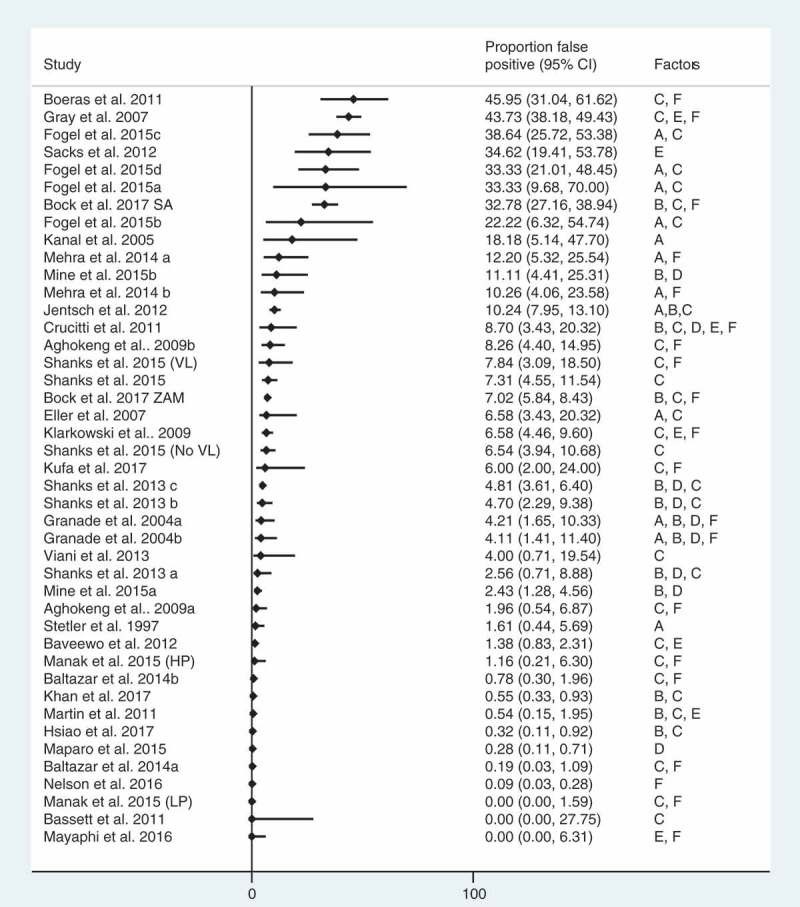
Rates of false positive diagnostic error rates diagnosis (*n* = 30 studies, 43 reports). LP: low prevalence; HP: high prevalence; ZAM: Zambia; VL: visceral Leishmaniasis; Data reported include reports of misdiagnosis of HIV-positive statuses. False positive diagnoses were reported in 30 studies (43 reports), total positive diagnoses *n* = 16,777. Kufa et al. 2017 reported proportion misdiagnosed by did not report full sample size. In studies where all participants were known to be HIV positive and/or on ART at the beginning of the study, the full study population was used as the denominator.

Thirty studies reported on false positive diagnostic errors (43 reports; *n* = 16,777 total positive diagnoses). In general, error rates were small (median: 3.1%; IQR: 0.4%-5.2%) with the exception of a few studies where a tiebreaker test was used to resolve discrepant results [10–14,23,32,33,37,39,41,44,46,47,75] (Figure 2). Of these, six studies (eight reports) exclusively among people with HIV enrolled in care or ART reported that between 0.1% (95% CI: 0–0.3) and 6.6% (95% CI: 4.5–9.6) of people were misdiagnosed (median: 1.6%, IQR: 0.3–4.7%) [10–13,33,37,39,41,47] (Table 3).

In studies reporting false positive diagnoses, nearly all reported the use of a suboptimal testing strategy [11–13,33,39,49,75]. Sixteen studies reported the use of a tiebreaker testing strategy were related to false positive HIV diagnoses [5,12,13,16,24,25,27,29,30,32,34,36,37,68]. In one of these studies, 95% (123/129) of false positive statuses resulted specifically from using a tiebreaker test [32]. Additionally, one study which reported misdiagnosis rates in Burundi, Democratic Republic of Congo (DRC) and Ethiopia reported some clients may have been provided an HIV-positive diagnosis based on a single HIV RDT [11].

### False negative diagnostic errors and misdiagnosis rates

**Figure 3.**
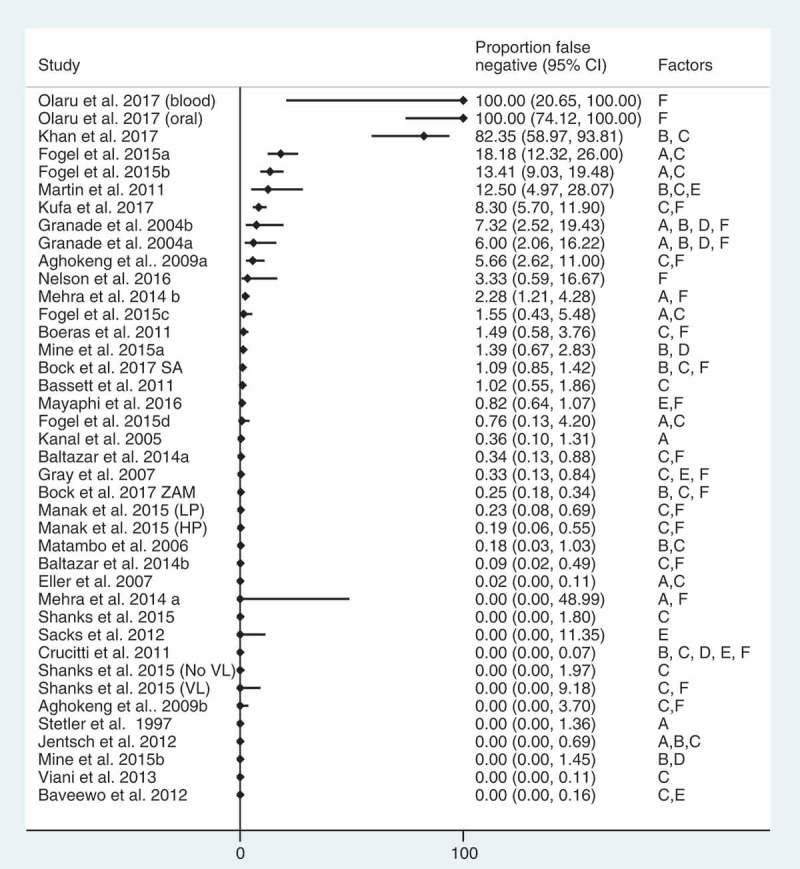
**False** **negative diagnostic error rates (*n* = 28 studies, 40 reports).** LP: low prevalence; HP: high prevalence; SA: South Africa, discrepant results; ZAM: Zambia, discrepant results; VL: visceral Leishmaniasis; Data reported includes reports of misdiagnosis of HIV-negative statuses. Misdiagnoses of HIV-negative statuses were reported 28 studies (40 reports), total negative = 55, 626. Kufa et al. 2017 reported proportion misdiagnosed but did not report full sample size information. Note Olaru et al. was exclusively among people with HIV on ART, accounting for the high rate of false negative diagnoses.

Twenty-eight studies reported on false negative diagnoses (40 reports, total negatives = 55,626) (median: 0.4%, IQR 0–3.9%) (Figure 3) [10,12,13,37,39,47,55–57]. The studies reporting the highest proportions, for example, Olaru et al., which was designed to assess how ART impacts test performance [58], of false negative diagnoses were exclusively among people with HIV on ART who were retested using an HIV RDT-based algorithm.

Nearly all studies reporting false negative diagnoses also reported using a suboptimal testing strategy. Four studies in South Africa reporting false negative diagnoses reported that HIV testing was conducted with an algorithm using a first-line test with high specificity and poorer sensitivity [14,33,39,55]. According to one of these studies [14], between 2014 and 2016, the testing algorithm changed four times in an effort to address the high proportion of false negative diagnoses resulting from these algorithms.

Clerical and user errors [57], early/acute infection [16,44,45], presentation late in disease stage [56] and individuals with known HIV status on ART who sought retesting, or were retested using oral fluid-based RDTs [58], were also reported as factors contributing to false negative diagnostic errors [14,16,39,44,47]. In Zimbabwe, all the reported false negative diagnoses were among children on ART who were retested with an oral fluid-based HIV RDT [58]. In South Africa and Zambia, individuals on ART comprised 44% (26/59) and 14% (5/38) of false negative diagnoses, respectively [14]. In Mozambique, 88% (21/24) of all true HIV-positive clients with a false negative test result were confirmed to know their HIV status and 62% (13/21) were reportedly on ART [47]; reasons for retesting in study reportedly included users misunderstanding the question or hoping to receive health services and emotional or mental health issues.

## Discussion

This review identified and described a number of diagnostic errors and poor HIV testing practices that may lead to misdiagnosis. Data on the magnitude of misdiagnosis was identified but limited, and no study could determine or quantify the exact cause(s) of misdiagnosis. Although no studies could determine and quantify the exact cause(s) of misdiagnosis, several identified the following factors to have strongly contributed: (1) suboptimal testing strategies, (2) poor management of supplies, (3) user errors including difficulty interpreting weak reactive lines and (4) retesting among people with known HIV status on ART.

No assay is perfect. False reactive and false non-reactive results are inevitable when using a single RDT and should be anticipated. However, the risk of misdiagnosis should be very low when a validated testing algorithm for high (≥5%) or low (<5%) prevalence settings is used [76]. In this review, we identified that many studies reporting diagnostic errors - both false positive and false negative - utilized suboptimal testing strategies which were not aligned to international guidance. Studies reviewed clearly showed the use of a tiebreaker strategy to rule-in HIV infection increases the likelihood of false positive statuses and possible misdiagnosis. This is concerning because a third of national testing strategies reviewed in 2015 recommended using a tiebreaker testing strategy [9].

In addition to adopting a proven testing strategy, national or regional validation is critical to determine which RDTs, and in which order, perform the best as a complete algorithm. As previously reported [38,77–83], tests and algorithm performance vary across settings, often due to cross-reactivity caused by HIV subtypes, co-infections, comorbidities and possible environmental or population characteristics. Without validating a testing algorithm at a country or regional level, it would not be possible to fully understand the causes of poor performance. Furthermore, to ensure correct diagnoses, it is important to retest people diagnosed HIV positive before they enrol in care and ART. This is a cost-effective approach [84] which is increasingly critical as more people with HIV are being offered immediate treatment.

To ensure correct results, all staff providing HIV testing must be trained, certified and provided ongoing support and supervision. In several studies, this was not the case, and untrained and uncertified providers were performing HIV testing [7,72]. Training, including pre-service, in-service and periodic refresher training, is important to maintain and improve the quality of services. Participation in EQA schemes is another way to monitor performance and improve testing services. Several studies also reported user and clerical errors resulted from inadequate support, demanding workloads, burnout and high levels of stress [11,62,64,66]. Adequate support and supervision are critical to reduce stock outs which may contribute to the use of damaged or expired test kits, incorrect test kits and buffer. Sites should routinely assess and manage their supplies and human resource planning to prevent or reduce these circumstances.

User error interpreting weak reactive lines was a common challenge which contributed to false positive results. To address this, specialized training for health workers and site-level standard operating procedures including the use of a “second-reader” to validate the correct interpretation of test results may be needed, as well as work with manufacturers to improve RDTs and instructions on how to interpret faint lines and weak control lines. Several studies hypothesized that weak reactive lines may be caused by other user errors, for example, misapplication of buffer and reading test results too early and cross-reactivity. Further investigation into the cause of weak reactive and other faint lines, and how they can be prevented, is needed.

False negative test results among people with HIV and on ART were observed and contributed to a substantial proportion of misdiagnoses [14,16,39,44,47]. While it is unclear why people on ART would seek retesting, some reports suggest it may be due to wanting to “check” or “confirm” one’s HIV status and religious beliefs about being “cured” [85], as well as misunderstandings and emotional or mental health issues [47]. It is important for programmes and users to be aware of the potential risk of false negative results, as the presence of ART can lead to confusing test results and could result in individuals unnecessarily stopping treatment which could have dire individual and public health implications. As “treat all” policies are rolled out, it will be increasingly critical for programmes to address this issue and ensure clients and health workers are aware that testing individuals on ART is not recommended [76].

## Strengths and limitations

This analysis is the first to bring together a diverse set of studies with the aim of identifying and describing suboptimal HIV testing practices and misdiagnosis. The results indicate the problem of misdiagnosis deserves attention. However, there are several limitations to this review.

As with all literature reviews, publication bias may be an issue and for this topic is inevitable and information on misdiagnosis is often unreported. This review was also limited to reports in English and may have missed reports in other languages. The majority of reports are from Africa and may not be representative of other geographies. Because the review was designed to identify reports of misdiagnosis, it is possible studies reporting errors and quality of HIV testing may have been missed.

Due to both the paucity and heterogeneity of data, it was not possible to conduct more quantitative analyses. Studies included were generally not designed to determine the exact cause or causes of misdiagnoses, a weakness cited across research on diagnostic errors [86].

This review focused on human errors and quality system failures. While we did identify some reports of cross-reactivity [10,22,24,25,27,32,38,56,60], reports did not provide conclusive information on what exactly caused cross-reactivity. Possible biological factors due to antibodies from inter-current infections, adverse environmental exposure to assay components, HIV subtype or shared false cross-reactivity in RDTs within an algorithm may be issues requiring further investigation.

Acute and early infection did not appear to be a significant cause of false negative diagnoses; however, few studies identified reported on acute infection. Retesting among HIV-positive individuals taking ART did emerge as a key factor contributing to a substantial proportion of false negative diagnostic errors and misdiagnoses. Further research is needed to understand how ART, as well as the use of antiretroviral drugs for prevention, for example, pre-exposure prophylaxis, may impact the performance of HIV RDTs, as well as how frequently people previously diagnosed with HIV and on ART retest.

## Conclusions

Our review has identified a number of factors and practices that may contribute to diagnostic error and HIV misdiagnosis. Although no study could fully determine and quantify the exact cause(s) of misdiagnosis, our review elucidated four key factors: (1) suboptimal testing strategies, primarily the use of a tiebreaker testing strategy to rule in HIV infection, (2) user errors including interpretation of weak reactive lines, (3) inadequate management and supervision of testers and (4) retesting among people with HIV on ART. Most, if not all, are avoidable with appropriate guidelines, training and supervision. The consequences of misdiagnoses are serious at an individual and public health level. With the momentum to scale-up HIV diagnosis and linkage to ART, a parallel push to improve the quality of HIV testing services and prevent misdiagnosis is essential.

## Notes

To access the supplementary material to this article please see Supplementary Files under Article Tools online.

## References

[1] WHO Towards universal access by 2010: how WHO is working with countries to scale-up HIV prevention, treatment, care and support. Geneva:World Health Organization;2006.

[2] WHO Factsheet to the WHO consolidated guidelines on HIV testing services. Geneva:World Health Organization;2015. 26378328

[3] UNAIDS Get on the fast-track: the life cycle approach to HIV. Geneva:Joint United Nations Programme on HIV/AIDS;2016.

[4] Molesworth AM, Ndhlovu R, Banda E, Saul J, Ngwira B, Glynn JR, et al High accuracy of home-based community rapid HIV testing in rural Malawi. J Acquir Immune Defic Syndr. 2010;55(5):625–18. 21934554 10.1097/QAI.0b013e3181f98628 PMC3248920

[5] Plate DK, Group, RHIVTEW Evaluation and implementation of rapid HIV tests: the experience in 11 African countries. AIDS Res Hum Retroviruses. 2007;23(12):1491–98. 18160006 10.1089/aid.2007.0020

[6] Lyamuya EF, Aboud S, Urassa WK, Sufi J, Mbwana J, Ndugulile F, et al Evaluation of simple rapid HIV assays and development of national rapid HIV test algorithms in Dar es Salaam, Tanzania. BMC Inf Dis. 2009;9. 10.1186/1471-2334-9-19 PMC2650699 19226452

[7] SEAD Analysis of POCT/VCT performed at South African primary health care clinics 2010 [cited 2014 7 14]. Available from: http://www.sead.co.za/downloads/POCT-clinics-2011.pdf

[8] Kyaw LL, Nozaki I, Wada K, Oo KY, Tin HH, Yoshihara N. Ensuring accurate testing for human immunodeficiency virus in Myanmar. Bull World Health Org. 2015;93(1):42–46. 25558106 10.2471/BLT.14.138909 PMC4271681

[9] Flynn D, Johnson C, Sands A, Wong V, Baggaley R Annex 2. An analysis of 48 national HIV testing and counselling policies. Geneva:World Health Organization;2015.

[10] Klarkowski DB, Wazome JM, Lokuge KM, Shanks L, Mills CF, O’Brien DP The evaluation of a rapid in situ HIV confirmation test in a programme with a high failure rate of the WHO HIV two-test diagnostic algorithm. PloS One. 2009;4(2). 10.1371/journal.pone.0004351 PMC2633037 19197370

[11] Shanks L, Klarkowski D, O’Brien DP False positive HIV diagnoses in resource limited settings: operational lessons learned for HIV programmes. PloS One. 2013;8(3). 10.1371/journal.pone.0059906 PMC3603939 23527284

[12] Shanks L, Ritmeijer K, Piriou E, Siddiqui MR, Kliescikova J, Pearce N, et al Accounting for false positive HIV tests: is visceral Leishmaniasis responsible? PloS One. 2015;10(7):e0132422. 26161864 10.1371/journal.pone.0132422 PMC4498794

[13] Shanks L, Siddiqui MR, Kliescikova J, Pearce N, Ariti C, Muluneh L, et al Evaluation of HIV testing algorithms in Ethiopia: the role of the tie-breaker algorithm and weakly reacting test lines in contributing to a high rate of false positive HIV diagnoses. BMC Infect Dis. 2015;15:39. 25645240 10.1186/s12879-015-0769-3 PMC4331460

[14] Bock P, Phiri C, Piwowar Manning E, Kosloff B, Mandla N, Young A, et al Understanding low sensitivity of community-based HIV rapid testing: experiences from the HPTN 071 (PopART) trial in Zambia and South Africa. J Int AIDS Soc. 2017;20(Suppl 6):21780. 28872272 10.7448/IAS.20.7.21780 PMC5625636

[15] WHO Diagnostic errors: technical series on safer primary care. Geneva:World Health Organization;2016.

[16] Manak MM, Njoku OS, Shutt A, Malia J, Jagodzinski LL, Milazzo M, et al Evaluation of performance of two rapid tests for detection of HIV-1 and −2 in high- and low-prevalence populations in Nigeria. J Clin Microbiol. 2015;53(11):3501–06. 26311857 10.1128/JCM.01432-15 PMC4609716

[17] Jaspard M, Le Moal G, Saberan-Roncato M, Plainchamp D, Langlois A, Camps P, et al Finger-stick whole blood HIV-1/-2 home-use tests are more sensitive than oral fluid-based in-home HIV tests. PloS One. 2014;9(6):e101148. 24971842 10.1371/journal.pone.0101148 PMC4074152

[18] O’Connell RJ, Merritt TM, Malia JA, VanCott TC, Dolan MJ, Zahwa H, et al Performance of the OraQuick rapid antibody test for diagnosis of human immunodeficiency virus type 1 infection in patients with various levels of exposure to highly active antiretroviral therapy. J Clin Microbiol. 2003;41(5):2153–55. 12734265 10.1128/JCM.41.5.2153-2155.2003 PMC154669

[19] Freeman MF, Tukey JW Transformations related to the angular and the square root. Ann Math Stat. 1950;21 :607–11.

[20] Miller J The inverse of the Freeman-Tukey double arcsine transformation. Amer Stat. 1978;32(4):138.

[21] Brown L, Cat T, DasGupta A Interval estimation for a proportion. Stat Sci. 2001;16:101–33.

[22] Aghokeng AF, Mpoudi-Ngole E, Dimodi H, Atem-Tambe A, Tongo M, Butel C, et al Inaccurate diagnosis of HIV-1 group M and O is a key challenge for ongoing universal access to antiretroviral treatment and HIV prevention in Cameroon. PloS One. 2009;4(11). 10.1371/journal.pone.0007702 PMC2768789 19893738

[23] Baltazar CS, Raposo C, Jani IV, Shodell D, Correia D, Da Silva CG, et al Evaluation of performance and acceptability of two rapid oral fluid tests for HIV detection in Mozambique. J Clin Micr. 2014;52(10):3544–48. 10.1128/JCM.01098-14 PMC4187771 25031435

[24] Baveewo S, Kamya MR, Mayanja-Kizza H, Fatch R, Bangsberg DR, Coates T, et al Potential for false positive HIV test results with the serial rapid HIV testing algorithm. BMC Res Not. 2012;5:154. 22429706 10.1186/1756-0500-5-154 PMC3392728

[25] Boeras DI, Luisi N, Karita E, McKinney S, Sharkey T, Keeling M, et al Indeterminate and discrepant rapid HIV test results in couples’ HIV testing and counselling centres in Africa. J Int AIDS Soc. 2011;14(1):18. 21477317 10.1186/1758-2652-14-18 PMC3086828

[26] CDC HIV testing for surveillance: evidence from assessments of routine diagnostic testing in ANC. Presented at: WHO HIV testing and counselling consultation; 2014 9 10-12; Geneva, Switzerland.

[27] Crucitti T, Taylor D, Beelaert G, Fransen K, Van Damme L Performance of a rapid and simple HIV testing algorithm in a multicenter phase III microbicide clinical trial. Clin Vac Immun. 2011;18(9):1480–85. 10.1128/CVI.05069-11 PMC3165239 21752945

[28] Da Costa Ferreira JO, Ferreira C, Riedel M, Widolin V, Ito S, Westman S, et al Field evaluation of an HIV rapid test algorithm for the rapid diagnosis of HIV infection in Brazil. Presented at: HIV Pathogenesis, Treatment and Prevention; 2007 7 22-25 Sydney, Australia.

[29] Eller LA, Eller MA, Ouma BJ, Kataaha P, Bagaya BS, Olemukan RL, et al Large-scale human immunodeficiency virus rapid test evaluation in a low-prevalence Ugandan blood bank population. J Clin Microbiol. 2007;45(10):3281–85. 17699650 10.1128/JCM.00894-07 PMC2045340

[30] Galiwango RM, Musoke R, Lubyayi L, Ssekubugu R, Kalibbala S, Ssekweyama V, et al Evaluation of current rapid HIV test algorithms in Rakai, Uganda. J Virol Methods. 2013;192(1–2):25–27. 23583487 10.1016/j.jviromet.2013.04.003 PMC3749432

[31] Granade TC, Parekh BS, Phillips SK, McDougal JS Performance of the OraQuick and Hema-Strip rapid HIV antibody detection assays by non-laboratorians. J Clin Virol. 2004;30(3):229–32. 15135740 10.1016/j.jcv.2003.12.006

[32] Gray RH, Makumbi F, Serwadda D, Lutalo T, Nalugoda F, Opendi P, et al Limitations of rapid HIV-1 tests during screening for trials in Uganda: diagnostic test accuracy study. BMJ Open. 2007;335(7612):188. 10.1136/bmj.39210.582801.BE PMC1934458 17545184

[33] Hsiao N, Zerbe A, Phillips T, Myer L, Abrams E Misdiagnosed HIV infection in pregnant women initiating universal ART in South Africa. J Int AIDS Soc. 2017;20(Suppl 6):21758. 10.7448/IAS.20.7.21758 PMC5625589 28872277

[34] Jentsch U, Lunga P, Lacey C, Weber J, Cairns J, Pinheiro G, et al The implementation and appraisal of a novel confirmatory HIV-1 testing algorithm in the Microbicides Development Programme 301 Trial (MDP301). PloS One. 2012;7(9). 10.1371/journal.pone.0042322 PMC3439440 22984401

[35] Kanal K, Chou TL, Sovann L, Morikawa Y, Mukoyama Y, Kakimoto K Evaluation of the proficiency of trained non-laboratory health staffs and laboratory technicians using a rapid and simple HIV antibody test. AIDS Res Ther. 2005;2:5. 10.1186/1742-6405-2-5 PMC1156864 15907202

[36] Karugaba P, Elbireer A, Nansamba A, Amukele T Weakly reactive HIV rapid diagnostic test kits shouldn’t be reported as positive. Presented at: Conference on Retroviruses and Opportunistic Infections; 2016 2 22–25; Boston, USA.

[37] Khan S, Mafara E, Pasipamire M, Spiegelman M, S; M, Ntshalintshali N, et al Identification of misdiagnosed HIV clients in an early access to ART for all implementation study in Swaziland. J Int AIDS Soc. 2017;20(Suppl 6):21756. 28872273 10.7448/IAS.20.7.21756 PMC5625592

[38] Klarkowski D, Glass K, O’Brien D, Lokuge K, Piriou E, Shanks L Variation in specificity of HIV rapid diagnostic tests over place and time: an analysis of discordancy data using a bayesian approach. PloS One. 2013;8(11). 10.1371/journal.pone.0081656 PMC3840056 24282615

[39] Kufa T, Kharsany A, Cawood C, Khanyile D, Lewis L, Grobler A, et al Misdiagnosis of HIV infection during a South African community-based survey: implications for rapid HIV testing. J Int AIDS Soc. 2017;20(Suppl 6):21753. 28872274 10.7448/IAS.20.7.21753 PMC5625550

[40] Learmonth KM, McPhee DA, Jardine DK, Walker SK, Aye TT, Dax EM Assessing proficiency of interpretation of rapid human immunodeficiency virus assays in nonlaboratory settings: ensuring quality of testing. J Clin Micr. 2008;46(5):1692–97. 10.1128/JCM.01761-07 PMC2395071 18353938

[41] Maparo T, Arhem J, Harrison R, Matimba M, Belaye AK, Bara H, et al An evaluation of false positive HIV results due to testing errors. Presented at: International Conference on AIDS and STIs in Africa; 2911 2015 Dec 4; Harare, Zimbabwe.

[42] Martin EG, Salaru G, Paul SM, Cadoff EM Use of a rapid HIV testing algorithm to improve linkage to care. J Clin Vir. 2011;52(Suppl. 1):S11–S5. 10.1016/j.jcv.2011.09.014 21983254

[43] Masina T Implementing HTS quality systems and retesting before ART initiation. Lilongwe:Ministry of Health Malawi;2017.

[44] Mayaphi SH, Martin DJ, Quinn TC, Laeyendecker O, Olorunju SA, Tintinger GR, et al Detection of acute and early HIV-1 infections in an HIV hyper-endemic area with limited resources. PloS One. 2016;11(10):e0164943. 27764165 10.1371/journal.pone.0164943 PMC5072595

[45] Mehra B, Bhattar S, Bhalla P, Rawat D Rapid tests versus ELISA for screening of HIV infection: our experience from a voluntary counselling and testing facility of a tertiary care centre in North India. Isrn Aids. 2014;2014:296840. 25006527 10.1155/2014/296840 PMC4004236

[46] Mine M, Chishala S, Makhaola K, Tafuma TA, Bolebantswe J, Merrigan MB Performance of rapid HIV testing by lay counselors in the field during the behavioral and biological surveillance survey among female sex workers and men who have sex with men in Botswana. J Acquir Immune Defic Syndr. 2015;68(3):365–68. 25394190 10.1097/QAI.0000000000000434

[47] Nelson R, MacKellar D, Thompson R, De Almeida M, Bonzela J, Mugabe D, et al Low prevalence of false prior HIV diagnoses in Chokwe district, Mozambique. Presented at: Conference on Retroviruses and Opportunistic Infections; 2016 2 22-25; Boston, USA.

[48] Sacks R, Omodele-Lucien A, Whitbread N, Muir D, Smith A Rapid HIV testing using Determine HIV 1/2 antibody tests: is there a difference between the visual appearance of true- and false-positive tests? Int J STD AIDS. 2012;23(9):644–46. 23033518 10.1258/ijsa.2012.011422

[49] Simoncini GM, Megill M, Berg-Wolf MV Reducing false-positive HIV diagnosis in Niger: a women’s issue. J Int Assoc Provid AIDS Care. 2015;15(1):15–8. 25979258 10.1177/2325957415586260

[50] Stetler HC, Granade TC, Nunez CA, Meza R, Terrell S, Amador L, et al Field evaluation of rapid HIV serologic tests for screening and confirming HIV-1 infection in Honduras. Aids. 1997;11(3):369–75. 9147429 10.1097/00002030-199703110-00015

[51] Tchounga BK, Inwoley A, Coffie PA, Minta D, Messou E, Bado G, et al Re-testing and misclassification of HIV-2 and HIV-1&2 dually reactive patients among the HIV-2 cohort of the West African Database to evaluate AIDS collaboration. J Int AIDS Soc. 2014;17(1):19064. 25128907 10.7448/IAS.17.1.19064 PMC4134669

[52] Wolpaw BJ, Mathews C, Chopra M, Hardie D, De Azevedo V, Jennings K, et al The failure of routine rapid HIV testing: a case study of improving low sensitivity in the field. BMC Health Serv Res. 2010;10:73. 20307310 10.1186/1472-6963-10-73 PMC2851712

[53] Viani RM, Araneta MRG, Spector SA Parallel rapid HIV testing in pregnant women at Tijuana General Hospital, Baja California, Mexico. AIDS Res Hum Retro. 2013;29(3):429–34. 10.1089/AID.2012.0190 23050550

[54] Young PW, Mahomed M, Horth RZ, Shiraishi RW, Jani IV Routine data from prevention of mother-to-child transmission (PMTCT) HIV testing not yet ready for HIV surveillance in Mozambique: a retrospective analysis of matched test results. BMC Infec Dis. 2013;13(1). 10.1186/1471-2334-13-96 PMC3598230 23432847

[55] Bassett IV, Chetty S, Giddy J, Reddy S, Bishop K, Lu Z, et al Screening for acute HIV infection in South Africa: finding acute and chronic disease. HIV Med. 2011;12(1):46–53. 20553336 10.1111/j.1468-1293.2010.00850.x PMC2970678

[56] Kahemele NJ, Lyyaruu E, Mayeye M HIV infection diagnostic uncertainity, a field experience.. Presented at: AIDS 2008 - XVII International AIDS Conference; 2008 3-8 8; Mexico City, Mexico.

[57] Matambo R, Dauya E, Mutswanga J, Makanza E, Chandiwana S, Mason PR, et al Voluntary counseling and testing by nurse counselors: what is the role of routine repeated testing after a negative result?. Clin Infect Dis. 2006;42(4):569–71. 16421803 10.1086/499954

[58] Olaru I, McHugh G, Dakshina S, Majonga E, Dauya E, Bandason T, et al False negative HIV tests using oral fluid tests in children taking antiretroviral therapy from Harare, Zimbabwe. J Int AIDS Soc. 2017. 10.7448/IAS.20.7.21751 PMC5625634 28872275

[59] Adebayo A, Olufemi A, Adekunle B, Louisa O A Limited survey on HIV-testing knowledge and practice of laboratory personnel at some point-of-care services in Abuja, Federal Capital Territory (FCT), Nigeria. Presented at: African Society of Laboratory Medicine; Cape Town, South Africa Oral Poster on Wednesday, 12. 5 2012 2012.

[60] Benzaken AS, Bazzo ML, Galban E, Pinto ICP, Nogueira CL, Golfetto L, et al External quality assurance with dried tube specimens (DTS) for point-of-care syphilis and HIV tests: experience in an indigenous populations screening programme in the Brazilian Amazon. Sex Trans Inf. 2014;90(1):14–18. 10.1136/sextrans-2013-051181 24031029

[61] Bile E, Bachanas P, Maurice F, Modise S, Chebani L, Makovore V, et al Accuracy of HIV and CD4 field testing in the Botswana combination prevention project. Presented at: Conference on Retroviruses and Opportunistic Infections; 13-16 2 2017; Seattle, USA.

[62] Cham F, Maleka M, Masango M, Goetsch E, Belabbes EH, Singh B, et al The World Health Organization African region external quality assessment scheme for anti-HIV serology. Afr J Lab Med. 2012;1(1). Art. 39. 10.4102/ajlm.v1i1.39 PMC5644520 29062735

[63] Iwe E, Livinus I, Omoregbe E, Okoye B, Nwuba C, Mbanefo A et al. Preparation and use of dry tube specimens (DTS) method by laboratorians and non- laboratorians for quality assurance of HIV services (HCT) in MSH supported sites, Niger State, North-Central Nigeria. Presented at: 6th IAS Conference on HIV Pathogenesis and Treatment; 2001 17-21 7; Rome, Italy.

[64] Kalou M, Mosha F, Ndlovu N, Jackson K, Mwassekage M, Ngoi M, et al Implementation of a proficiency testing program using dried tube specimen (DTS) approach to improve the quality of HIV rapid testing in testing sites in Uganda and Tanzania. Presented at: First International Conference of the African Society for Laboratory Medicine; 2012 512 2012; Cape Town, South Africa.

[65] Kitheka F, Umuro M, Mwa S, editors. Implementing rapid HIV proficiency testing program in Kenya: key findings from corrective actions following the switch from facility-based to individual-based proficiency testing. First International Conference of the African Society for Laboratory Medicine; 2012 512 2012; Cape Town, South Africa.

[66] Louis FJ, Anselme R, Ndongmo C, Buteau J, Boncy J, Dahourou G, et al Evaluation of an external quality assessment program for HIV testing in Haiti, 2006-2011. Am J Clin Pathol. 2013;140(6):867–71. 24225755 10.1309/AJCPYWX49IZSQKFS PMC4733348

[67] Lali W, Guma G, Awongo P, Akol Z, Kajumbula H, Namupijja P, et al Challenges of implementation of integrated national laboratory quality improvement in Uganda. Presented at: AIDS 2010 - XVIII International AIDS Conference; 2008 18–23 7; Vienna, Austria.

[68] Manyazewal T, Paterniti AD, Redfield RR, Marinucci F Role of secondary level laboratories in strengthening quality at primary level health facilities’ laboratories: an innovative approach to ensure accurate HIV, tuberculosis, and malaria test results in resource-limited settings. Diagn Microbiol Infect Dis. 2013;75(1):55–59. 23102548 10.1016/j.diagmicrobio.2012.09.020

[69] Mashauri FM, Siza JE, Temu MM, Mngara JT, Kishamawe C, Changalucha JM Assessment of quality assurance in HIV testing in health facilities in Lake Victoria zone, Tanzania. Tanzan Health Res Bull. 2007;9(2):110–14. 17722413 10.4314/thrb.v9i2.14312

[70] Mwangala SM, Musonda K, Monze M, Musukwa K, Fylkesnes K Accuracy in HIV rapid testing among laboratory and non-laboratory personnel in Zambia: observations from the National HIV Proficiency Testing System. Int J Inf Dis. 2014;21:129. 10.1371/journal.pone.0146700 PMC4706302 26745508

[71] Ntim NAA, Nyarko KM Quality audit of rapid HIV diagnostic processes and outcomes in selected health facilities in the central region of Ghana. Int J Inf Dis. 2014;21:132.

[72] Ocheng D, Mattaro S, Msilama L, Msauka A, Khama M, Mshanga N, et al Establishing competence in HIV rapid testing among non-laboratory health professional is key to tasking: preliminary results from ten regions of Tanzania. Presented at: 19th International AIDS Conference; 2012 22-27 7; Washington, DC.

[73] Sushi K, Saraminijacob G, Thatchinamoorthy G External quality assurance scheme in a national reference laboratory for HIV testing in resource limited settings, India. Presented at: 6th IAS Conference on HIV Pathogenesis and Treatment; 2001 17-21 7; Rome, Italy.

[74] Tegbaru B, Wolday D, Messele T, Meless H, Kassa D, Tesema D, et al Assessment of the implementation of HIV-rapid test kits at different levels of health institutions in Ethiopia. Ethiop Med J. 2007;45(3):293–99. 18330330

[75] Fogel JM, Piwowar-Manning E, Donohue K, Cummings V, Marzinke MA, Clarke W, et al Determination of HIV status in African adults with discordant HIV rapid tests. J Acquir Immune Defic Syndr. 2015;69(4):430–38. 25835607 10.1097/QAI.0000000000000610 PMC4483143

[76] WHO Consolidated guidelines on HIV testing services. Geneva:World Health Organization;2015. 26378328

[77] Lejon V, Ngoyi DM, Ilunga M, Beelaert G, Maes I, Buscher P, et al Low specificities of HIV diagnostic tests caused by *Trypanosoma brucei* gambiense sleeping sickness. J Clin Microbiol. 2010;48(8):2836–39. 20573878 10.1128/JCM.00456-10 PMC2916589

[78] Esteva MH, Blasini AM, Ogly D, Rodriguez MA False positive results for antibody to HIV in two men with systemic lupus erythematosus. Ann Rheum Dis. 1992;51(9):1071–73. 1417140 10.1136/ard.51.9.1071 PMC1004841

[79] Riberio T, Brites C, Moreira EJ, Siller K, Silva N, Johnson WJ, et al Serologic validation of HIV infection in a tropical area. J Acquir Immune Defic Syndr. 1993;6(3):319–22. 8450408

[80] Everett DB, Baisely KJ, McNerney R, Hambleton I, Chirwa T, Ross DA, et al Association of schistosomiasis with false-positive HIV test results in an African adolescent population. J Clin Micr. 2010;48(5):1570–77. 10.1128/JCM.02264-09 PMC2863920 20181896

[81] Salinas A, Górgolas M, Fernández-Guerrero M Refrain from telling bad news: patients with Leishmaniasis can have false-positive HIV test results. Clin Inf Dis. 2007;45(1):139–40. 10.1086/518709 17554722

[82] Klarkowski D, O’Brien DP, Shanks L, Singh KP Causes of false-positive HIV rapid diagnostic test results. Exp Rev Anti Inf Ther. 2014;12(1):49–62. 10.1586/14787210.2014.866516 24404993

[83] Kosack CS, Page AL, Beelaert G, Benson T, Savane A, Ng’ang’a A, et al Towards more accurate HIV testing in sub-Saharan Africa: a multi-site evaluation of HIV RDTs and risk factors for false positives. J Int AIDS Soc. 2017;19(1):1–12. 10.7448/IAS.20.1.21345 PMC5467586 28364560

[84] Eaton J, Johnson C, Gregson S The cost of not retesting: human immunodeficiency virus misdiagnosis in the antiretroviral therapy “test-and-offer” era. Clin Infect Dis. 2017;cix341. 10.1093/cid/cix341 PMC5850410 28444206

[85] Kumwenda M, Munthali A, Phiri M, Mwale D, Gutteberg T, MacPherson E, et al Factors shaping initial decision-making to self-test amongst cohabiting couples in urban Blantyre, Malawi. AIDS Behav. 2014;373(26):2491–2493. 10.1007/s10461-014-0817-9 PMC4102820 24929834

[86] Khullar D, Jha A, Jena A Reducing diagnostic errors – why now? N Eng J Med. 2015:2491–93. 10.1056/NEJMp1508044 PMC4943217 26397948

